# P201: First report in the world of Mycobacterium bacteremicum causing a cluster of postlaparotomy surgical wound infections

**DOI:** 10.1186/2047-2994-2-S1-P201

**Published:** 2013-06-20

**Authors:** M Biswal, G Singh, V Jain, SB Appannanavar, V Hallur, PK Gupta, S Malvi, N Taneja, S Sethi

**Affiliations:** 1 Department of Medical Microbiology, Chandigarh, India; 2 Postgraduate Institute of Medical Education and Research, Chandigarh, India

## Introduction

Uncommon atypical mycobacteria previously known to be environmental contaminants are an increasingly reported cause of outbreaks of surgical site wound infections. We investigated a cluster of post-laparotomy wound infections in 12 patients at our tertiary care hospital in India. We describe the epidemiology and the methods used to investigate the outbreak.

## Objectives

The objective of this study was to investigate a cluster of post-laparotomy wound infections in 12 patients at our tertiary care hospital in India using 16SrRNA typing.

## Methods

The outbreak started in October, 2011 and continued till April, 2012. The patients presented with delayed wound healing post laparotomy surgery. Swabs collected from the gaping wounds were sent for culture of atypical mycobacteria. Samples were also collected from the environment to locate the source of the organism. Samples were plated on Middlebrook 7H10 and Lowenstein-Jensen medium. *Mycobacteria* were identified by partial 16S r RNA sequencing.

## Results

All specimens yielded a yellow pigmented rapidly growing mycobacterium species. The sequences (Seq1 and Seq2) obtained by PCR using 16S rRNA PCR were compared with that in the GenBank database. The sequences of our isolates gave 99% identity with the ex-type strain of *Mycobacterium bacteremicum* (ATCC 25791). Sequence alignment and phylogenetic tree were constructed using the neighbour-joining method with MEGA5.1software package. Sequence data were submitted to the GenBank (Accession No. JX473587 & JX473588).

## Conclusion

In conclusion, delayed wound healing in surgical patients should be investigated for atypical mycobacteria using molecular methods to reach a diagnosis and institute appropriate and prolonged antimicrobial treatment. To the best of our knowledge, the causative agent, *M. bacteremicum* is being reported to cause post-surgical wound infection for the first time in world literature.

## Disclosure of interest

None declared

